# Radium and Lead Radioisotopes Composition of Sediment and Its Biogeochemical Implication in Polymetallic Nodule Area of Clario-Clipperton Zone

**DOI:** 10.3390/molecules27165061

**Published:** 2022-08-09

**Authors:** Feng Lin, Cai Lin, Wen Yu, Xiuwu Sun, Hui Lin

**Affiliations:** 1 Third Institute of Oceanography, Ministry of Natural Resources, Xiamen 361005, China; 2 School of National Safety and Emergency Management, Beijing Normal University, Zhuhai 519087, China

**Keywords:** radioactivity, ^210^Pb, ^226^Ra, bioturbation, sediment core

## Abstract

Radioactivity levels of ^210^Pb and ^226^Ra were detected in a sediment core obtained using a multi-corer from the polymetallic nodule area inside the Clarion-Clipperton Zone (CCZ), a contract area of the China Ocean Mineral Resources Association (COMR) in the eastern Pacific Ocean. The profile of excess ^210^Pb (^210^Pb_ex_) shows that the specific activity of ^210^Pb_ex_ has three parts with different distributions at depths of 0–16 cm (I), 17–36 cm (II), and 37–48 cm (III). When the I section of nonlocal mixing was excluded, using a steady-state diffusion mode, the bioturbation coefficients of the core were estimated to be 24.2 cm^2^/a at 17–36 cm deep and 5.9 cm^2^/a at 37–48 cm deep, which were greater compared to previously published results. This is most likely owing to bioturbations caused by various organism species in the two sections.

## 1. Introduction

There are approximately 54 × 10^6^ km^2^ of polymetallic nodules in the global oceans, of which the largest area is in the Pacific Ocean, with approximately 23 × 10^6^ km^2^, especially in the Clarion-Clipperton Zone (CCZ) of the eastern Pacific Ocean, which is the most abundant and potentially most economically valuable [1]. The estimated reserves of polymetallic nodules in the CCZ are approximately 21 × 10^9^ t, including approximately 6 × 10^9^ t of Mn, which is larger than all known terrestrial Mn reserves. Meanwhile, the Ni content (270 Mt) and Co content (44 Mt) of polymetallic nodules in the CCZ are three and five times higher than the terrestrial reserves, respectively [2]. With the expansion of the global economy, all nations face a significant resource dilemma, and deep-sea mineral resources dispersed in international public seas are gaining attention from governments and organizations throughout the world.

Traditionally, the primary diagenetic processes of deep-sea polymetallic nodules have been diagenesis and hydrogenesis, but the relevance of biomineral production of deep-sea polymetallic nodules has garnered widespread attention in recent years [3,4,5]). Furthermore, benthic fauna plays a significant role in the production of both polymetallic nodules and Corich crusts. It is acknowledged that mining and future exploitation would erode the hard substrate and bottom deposition, and may generate sediment plumes, potentially impacting benthic faunal ecosystems across broad regions. Although seabed habitats are particularly vulnerable, data on density, richness, and community structure are missing in many sections of the CCZ [6].

^210^Pb is a naturally occurring radioisotope in the uranium decay series that has been regularly used to assess sedimentation rates and biological mixing records during the last 100 years. The gaseous decay product of its parent ^226^Ra, ^222^Rn, escapes from land and decays through a sequence of short-lived daughter bodies to produce ^210^Pb. ^210^Pb remains in the atmosphere for a brief period of time (about 10 days) before migrating out of the sky via rain, snow, dust, etc. [7,8], and settling on the ocean surface before settling into the sediment, resulting in ^210^Pb having higher specific activity than its parent ^226^Ra [9]. Sinking particles convey ^210^Pb to the bottom, which is produced by the disintegration of ^226^Ra and ^222^Rn in the atmosphere and water column. The ^210^Pb incorporated into the sediment as a result of this process is known as “excess ^210^Pb”. The ^210^Pb_ex_ signal decays with time, eventually approaching zero after around 100 years. If sediment accumulation alone affects ^210^Pb_ex_ distribution, the extra ^210^Pb signal will be unnoticeable after 100 years, the excess ^210^Pb relevant to ^226^Ra indicates a distinct mixing process in the sediments, which was caused by benthic activity [10].

The purpose of this study was to estimate the bioturbation coefficients at the Polymetallic Nodule Area of CCZ based on high-resolution radionuclide activity distributions in the sediment core.

## 2. Materials and Methods

### 2.1. Sampling

**Figure 1:**
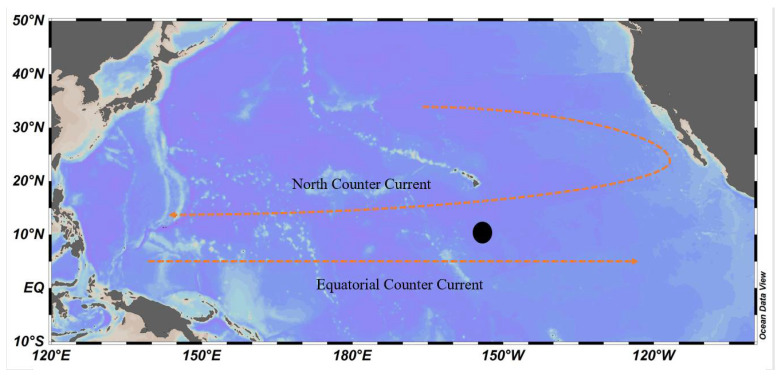
Sampling locations.

On the Monitoring and Protection of Ecology and Environment Cruise in the northeast Pacific in 2017, a sediment core KW1-S05-MC13 was collected on the R/V XIANGYANGHONG03 (Third Institute of Oceanography, MNR, Xiamen, China) (Figure 1, Table 1). Which was discovered at a bottom depth of 5167 m at the sampling site (10.0833° N, −154.3334° E) in the China Ocean Mineral Resources Association’s (COMR) northern polymetallic nodule exploration contract region.

The sediment sample was taken using a multi-core sampler with an inner diameter of 9.5 cm, which may gather many undisturbed sediment samples from the seafloor with a deploy, including the sediment-water interface and benthic life. Within 24 h of being collected, the sediment cores were extruded and sectioned at 1 cm intervals. Each subsample was packed individually in a clean polyethylene bag and kept frozen at −18 °C. The samples were sent to a land-based laboratory for examination.

### 2.2. ^210^Pb and ^226^Ra Analysis

The freeze-dried materials were crushed, pulverized, sieved, and homogenized before being powdered. Due to the detector shield’s restricted size, about 2–3 g of the samples was moved to cylindrical plastic containers for gamma spectrometric counting. The cylindrical plastic canisters were sealed for roughly 20 days prior to gamma counting to allow radon and its short-lived progeny to attain secular radioactive equilibrium.

One lead-shielded high-purity germanium (HPGe) detector (Model 9030, Ortec Inc., Atlanta, GA, USA), with an efficiency of 20% and a full width at half maxima (at 1332 keV) less than 2.1, was used for the gamma spectrometry. The characteristic gamma ray used in ^210^Pb counting is 46.5 keV. The specific radioactivity of ^210^Pb was calculated according to Formula (1). A self-absorption correction for low-energy ^210^Pb γ rays was performed following the method described by Cutshall et al. (1983) [11]:

(1) $$A=\overset{n}{\underset{i=1}{\sum }}\left(\frac{N_{i}}{t}-\frac{N_{bi}}{t_{b}}\right)\times \frac{1}{\varepsilon_{i}Y_{i}m}$$

where A is the radioactivity of ^210^Pb (Bq/kg); *N_i_* and *N_bi_* are respectively the counts of the peaks at 46.5 keV of the sample and the blank; *t* and *t_b_* are the counting times for the sample and blank, respectively; $\varepsilon_{i}$ is the detecting efficiency of ^210^Pb calibrated with the marine sediment reference material produced by the International Atomic Energy Agency (IAEA-385); *m* is the mass of the sample (kg); and *Y* is branch ratio (4.25%).

The radioactivity of ^226^Ra was derived from the radioactivity levels of its daughter isotopes ^214^Pb and ^214^Bi, assuming that they reached equilibrium in the 3-week storage prior to counting. The energy transitions are 295.2 keV (18.4%) of ^214^Pb and 609.3 keV (45.5%) of ^214^Bi. The HPGe spectrometer was calibrated for the ^226^Ra quantitative analysis with the sediment reference material (GBW08304a, produced by the National Center of Reference Material, Beijing, China) containing a known amount of ^226^Ra.

The acquisition time for the spectrum and the blank were 72 h and 120 h, respectively. All the reported activity values were decay corrected to the date of sample collection.

The excess ^210^Pb (^210^Pb_ex_), which is the fraction of ^210^Pb activity exceeding the activity of the parent isotope ^226^Ra, was determined by subtracting the activity of ^226^Ra from the activity of ^210^Pb.

### 2.3. Quality Controls

10% to 15% of the samples were selected for parallel determination. All the standard deviation of the parallel results was within the permissible range (15%), and then the average of the results was taken as the final result for the specific sample.

Besides, the laboratory has participated in the annual environmental radionuclides analysis proficiency tests organized by the International Atomic Energy Agency (IAEA) and achieved good results, demonstrating that the research team has good quality control in radionuclide measurements.

## 3. Results

### The Distribution of ^226^Ra and ^210^Pb

**Figure 2:**
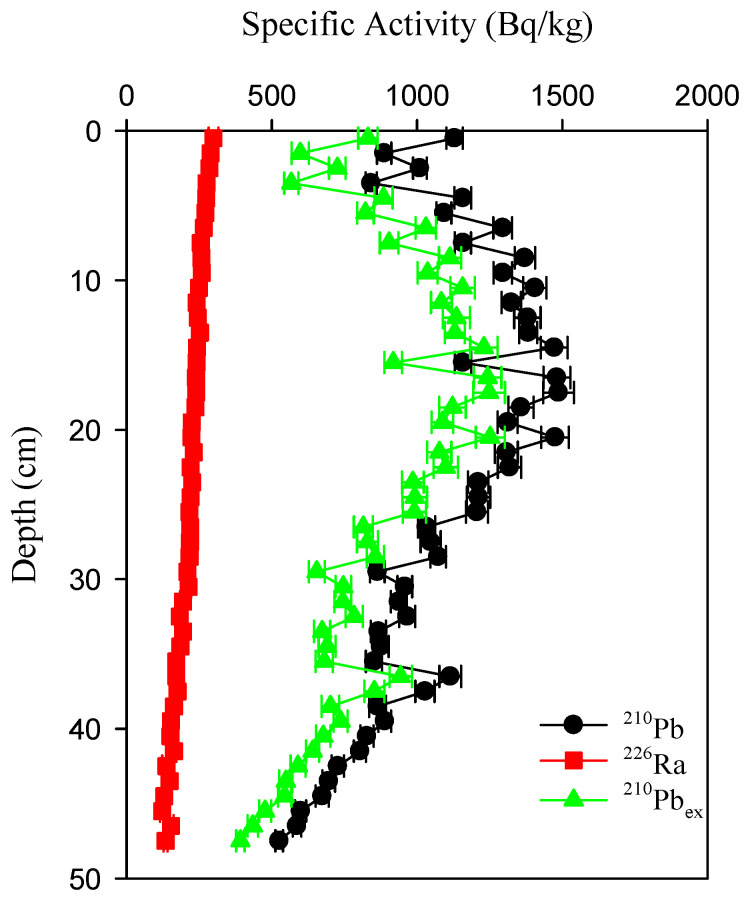
Profiles of ^210^Pb, ^226^Ra, and ^210^Pb_ex_ specific activity in the sediment core.

The profiles of ^226^Ra and^210^Pb specific radioactivity are given in Figure 2. The specific radioactivity of ^226^Ra varied from 298 Bq/kg at the surface to 122 Bq/kg at the bottom, with an average of 214 ± 47 Bq/kg. (The value after the “±” sign is the uncertainty of the measured result, which depends on the net counting rate obtained in the measuring process and is a characteristic of radioactivity measurement result). The profiles of ^226^Ra-specific radioactivity in the core decreased gradually with the depth.

The specific radioactivity of ^210^Pb varied from 525 Bq/kg to 1488 Bq/kg, with an average of 1074 ± 263 Bq/kg. It increased with increasing depth and then descended in an exponential manner with the depth after reaching a maximum at a depth of 18 cm.

## 4. Discussion

### 4.1. The Radioactive Disequilibrium between ^226^Ra and ^210^Pb

^210^Pb created in the atmosphere and water column by the decay of ^226^Ra and ^222^Rn, was transported on particles to the seafloor by sinking. The ^210^Pb integrated into the sediment as a result of this process is referred to as “excess ^210^Pb”, since it exists in excess of the ^210^Pb created in situ by the decay of ^226^Ra and ^222^Rn within the sediment column (“supported ^210^Pb”).

The activity of ^210^Pb supported by ^226^Ra is substantially regulated by the activity of ^230^Th, ^226^Ra’s parent. Scavenging from the water column and ingrowth from ^234^U, which is initially in excess due to the high U content of these aragonite-rich sediments, influence ^230^Th activities in turn.

The main source of ^210^Pb in deep-sea sediments is the decay of the parent ^226^Ra. If there are no other processes, the activity distributions of ^210^Pb and ^226^Ra should be similar. However, the showed results indicate that the activity of ^210^Pb did not change with ^226^Ra. The difference between the two radionuclides indicates that ^210^Pb is not entirely from the production of ^226^Ra in the sediment. At the same time, there is a significant excess of ^210^Pb in the whole column.

The specific radioactivity of ^210^Pb_ex_ ranged from 391 Bq/kg to 1252 Bq/kg, with an average of 860 ± 234 Bq/kg. It gradually increases with depth, and reaches a maximum of 20 cm. Then it gradually decreases with depth. But there is an uptick at 36–40 cm.

The significant excess of ^210^Pb further confirms other sources of ^210^Pb in the surface sediments. Combined with the strong particle activity of ^210^Pb, the excess ^210^Pb should come from ^210^Pb removed by particulate matter in the bottom water, which would result in a deficit of ^210^Pb relative to ^226^Ra in the bottom water. Elevated particulate ^210^Pb in the Pacific Ocean bottom water supports a stronger particle-scavenging ^210^Pb process in the bottom water [12,13].

Although the ^210^Pb removed from the bottom water settled on the sediment surface, a significant excess of ^210^Pb was still observed in the entire column below the surface to a depth of 48 cm. The sedimentation rate in the vicinity of the study sea area was less than 1 cm/ka. If there is no mixing process, with a half-life of only 22.3 a for ^210^Pb, it should reach equilibrium with ^226^Ra within 1 mm of the surface layer. The actual observations were the opposite, revealing a distinct mixing process in the sediments, which was caused by benthic activity.

Smith and Schafer (1984) used a “conveyor belt” hybrid model to explain the existing ^210^Pb subsurface sediment maximum value distribution in the North-East Atlantic 4000–5000 m depth [14]; specifically, the benthos feeding is rich in organic matter and radioactive nuclide surface sediment after its discharge to the surface sediment, and the recent single suddenly mixed event model results were consistent with the measured radionuclide section. Smith et al. (1997) discovered ^210^Pb_ex_ in deep ocean sediments of the equatorial Pacific Ocean, which displayed a “shoulder phenomenon” at a depth of 23 cm, which researchers suspected was caused by the burrowing operations of huge sea urchins [13]. The equatorial Pacific Ocean’s bottom sediments are densely packed with sea urchins. Sea urchins frequently excavate trenches a few decimeters wide, and they disrupt the surface sediments in these locations every 5 to 7 years [15].

The second largest value of ^210^Pb_ex_ in sediments from various Peruvian Sea basin sites emerged at depths of 5–20 cm (the first maximum value was at the surface), which was thought to be generated by surface eating and subsurface excretion of animals such as Echinaceae [16]. The Yi insects are responsible for the tropical Pacific Ocean sediments in northeast ^210^Pb_ex_ at 22 to 28 cm and 6–10 cm in the subsurface peak, and they rotated the trail of the in situ hybrid [17].

### 4.2. Bioturbation Coefficient

The quantification of bioturbation is required for the numerical modeling of sediment diagenesis. Using a steady-state diffusion mode, the bioturbation coefficient (*D_B_*) was calculated from the ^210^Pb_ex_ activity profile in the sediment. Because of the simple and fast sample measurement, the simultaneous measuring of ^226^Ra and ^210^Pb in the sample is capable. Thus the steady-state diffusion mode has been widely applied in studies of sediment bioturbation. In this model, bioturbation and sedimentation are considered to be similar to diffusion and advection. The biological mixing process of tracer material in sediments must fulfill two characteristics when a biological diffusion model is used: (1) The frequency of biological mixing must be significantly larger than the tracer’s disappearance rate, and (2) the particle exchange size must be less than the size of the tracer profile and the thickness of the mixing layer.

Since the half-life of ^210^Pb is 22.3 a, ^210^Pb will reach a radioactivity equilibrium with ^226^Ra in five half-lives (~100 years). Therefore, the ^210^Pb_ex_ signal will appear in the sediment that accumulated in the recent period of approximately 100 years. Considering that the sedimentation rates in the study area are estimated to be lower than 1 mm per thousand years, ^210^Pb_ex_ should be detectable only within the top 1 mm of the sediment core, if there is no bioturbation or other disturbance. However, in the core samples collected in this study, ^210^Pb_ex_ signal was found down to a depth of dozens of centimeters, which was interpreted to be a result of bioturbation. To represent the process of bioturbation, Guinasso and Schink (1975) and Nozaki (1977) devised and modified a steady-state diffusion model [18,19]:

(2) $$\frac{\partial }{\partial t}\left(\rho A\right)=\frac{\partial }{\partial z}\left(\rho D_{B}\frac{\partial A}{\partial z}\right)-\frac{\partial }{\partial z}\left(\rho SA\right)-\lambda \rho A$$

where *z* is the depth of the sediment sample (cm), A is the ^210^Pb_ex_ radioactivity (Bq/kg) at the depth of *z*, *ρ* is the density (g/cm^3^), *D_B_* is the bioturbation coefficient (cm^2^/a), *S* is the sedimentation rate (cm/a), λ is the decay constant of ^210^Pb (0.031 a^−1^) and t is the time (a).

Assuming that in the mixing layer, *D_B_*, *S* and ρ are in a steady state, then $\;\frac{\partial }{\partial t}\left(\rho A\right)=0$.

With the boundary conditions: A = A_0_ at *z* = 0; and (2) A→0 at *z*→∞:

(3) $$A=A_{0}exp\left[\frac{S-\sqrt{S^{2}+4\lambda D_{B}}}{2D_{B}}z\right]$$

Since *S* for oceanic sediments is on the order of mm/ka and the valid time scale of applying ^210^Pb in tracing bioturbation is approximately 100 a, the sediment deposition process can be ignored, so Equation (3) is simplified as:

(4) $$A=A_{0}exp\left[-z\sqrt{\lambda /D_{B}}\right]$$

Therefore, the exponential fit of the measured profile of ^210^Pb_ex_ quantifies the process of bioturbation.

**Figure 3:**
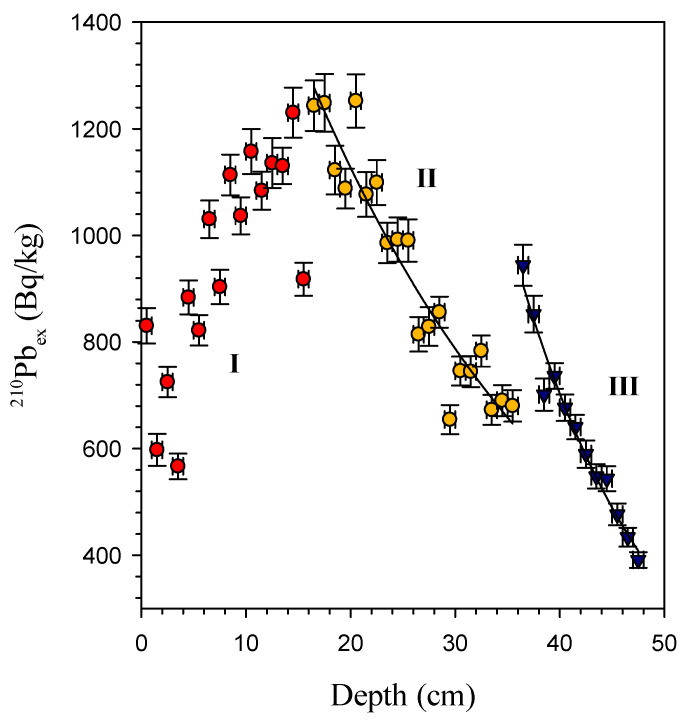
Radioactivity of ^210^Pb_ex_ against depth in core samples.

The depth resolution of radiotracers in this study is very high compared to other research [20,21,22]. The profile of ^210^Pb_ex_ shows that the specific activity of ^210^Pb_ex_ has three different distributions at depths of 0–16 cm (I), 17–36 cm (II), and 37–48 cm (III), indicating that the bioturbation coefficient has changed (Figure 3). This suggested that the particle mixing process should be complicated. The complicated vertical profiles were also observed in the Pacific [22,23]. The maximum value in the subsurface layer indicates heterogeneous mixing of particles by benthic organisms [14,24,25]. The ^210^Pb_ex_ data affected by nonlocal mixing are excluded when estimating *D_B_.*

The calculated *D_B_* are 24.2 cm^2^/a and 5.9 cm^2^/a in sections II and III, respectively. The *D_B_* values of our research are significantly higher than Yang and Zhou (2004) (0.26–2.75 cm^2^/a) [15] and Hyeong et al. (2018) (1.1–9.0 cm^2^/a) [20]. On the other hand, it is clear that the *D_B_* of section II is higher than that of section III. The *D_B_* of section III is the same as Hyeong et al. (2018) [20]. This is most likely owing to bioturbations caused by various organism species in the two sections. This will necessitate further investigation in the future.

In the meantime, it should be reminded that it is not reasonable to portray the entire area with the bioturbation coefficients estimated with a single sediment core sample. Although obtaining sediment core from the deep ocean is very cost-consuming, further investigation is needed in the future to better understand the characteristics of this important region.

## 5. Conclusions

The radioactivity profiles of ^210^Pb and ^226^Ra in sediment core from CCZ were measured. A significant ^210^Pb_ex_ signal was found throughout the core and has three parts with different distributions at depths of 0–16 cm (I), 17–36 cm (II), and 37–48 cm (III). Based on the radioactivity profiles of ^210^Pb_ex_ in sediment cores from CCZ and the one-dimensional steady-state diffusion model, bioturbation coefficients at one station of different sections were estimated to be 24.2 cm^2^/a at 17–36 cm deep and 5.9 cm^2^/a at 37–48 cm deep, which are larger than the reported values, indicating that this region is biologically abundant and has evident bioturbation processes. This is most likely owing to bioturbations caused by various organism species in the two sections. This will necessitate further investigation in the future.

## Figures and Tables

**Table 1:** Coordinates of sampling stations and bioturbation coefficients.

Station	Latitude (°N)	Longitude (°E)	Bottom Depth (m)	D_b_ (cm^2^/a)
KW1-S05-MC13	10.0833	−154.3334	5167	24.2 (II)
				5.9 (III)

## Data Availability

The data presented in this study are available on request from the corresponding author.
